# Educational Assortative Mating and Health: A Study in Chinese Internal Migrants

**DOI:** 10.3390/ijerph18041375

**Published:** 2021-02-03

**Authors:** Ling Zhang, Xiaodong Tan

**Affiliations:** School of Health Sciences, Wuhan University, Wuhan 430071, China; zhangling0@whu.edu.cn

**Keywords:** marriage, education, assortative mating, health, mate selection

## Abstract

Previous studies have shown that marriage is related with people’s health. Based on data from the Volume A of China Migrants Dynamic Survey (CMDS_A) in 2017 (N = 127,829), this study attempted to document the degree of educational assortative mating in Chinese internal migrants, as well as how it evolves over time, and further analyze the relationship between educational assortative mating and people’s self-rated health (SRH). The results indicated that the proportion of educational homogamy kept increasing and gradient marriage kept decreasing over time both in male and female. “Educational homogamy” (58.8%) and “male more educated” (27.2%) were still the main marital education matching patterns in first-married couples of Chinese internal migrants. Educational homogamy was beneficial to promote people’s SRH and educational hypogamy would impair their SRH, and the negative effects of educational hypogamy on SRH was stronger in female than in male. The gender equality of educational opportunities increases the degree of educational assortative mating in Chinese internal migrants. Educational attainment is playing a more and more important role in “love” marriages. “Likes attract likes” is not just about love, but also an important part of health.

## 1. Introduction

### 1.1. Assortative Mating and Chinese Internal Migrants

Assortative mating is defined as the nonrandom coupling of individuals based on their similarity to each other on one or more characteristics [1]. Previous evidence shows that people are more inclined to choose a mate who is similar to himself/herself [2,3]. The economic development brought by labor mobility and urbanization makes the choice of marriage more diversified and the mobility of socioeconomic stratum more frequent [4]. Large-scale social transition in China has produced numerous internal migrants, mainly young and middle-aged, number of which has reached 244 million by the end of 2017 [5]. Moving from hometown to other areas, changes of living circumstance may bring changes in their concept of marriage and mate selection criteria. The change of social circle would change their scope of mate selection as well.

### 1.2. Migration and Health

As an indispensable part of China’s labor force, internal migrants have made considerable contributions to the development of China. Nevertheless, there are some institutional and structural constraints in their process of migration, such as *hukou* (*Hukou* is a unique household registration system in China brought by traditional urban-rural dual social structure. It is important for individuals in securing access to social welfare such as health care) restrictions, bringing them higher health risks. Health problems of internal migrants are closely related to their low socio-economic status and other factors. Numerous evidence suggests that internal migrants’ health will suffer over time as they usually experience more specific unhealthy exposures during their migration [6,7]. Social support is essential for Chinese internal migrants’ health. Marriage is considered to be the main source of social support and positively related to health [8].

### 1.3. Literature Review

As a kind of social relationship, marriage is an important part of human social life. Mating is the behavior of men and women choosing whom to marry by their own choice. From the macro level, mating is related to the sex ratio in the whole marriage market, socio-cultural traditions and the policies implemented. From the micro-level, mating is also related to the individual’s psychology and inclination in spouse selection, social status, educational level and so on [9]. Normally, people will consciously gather into a certain group according to their country, educational background, occupation, family background and so on. Individuals in similar social groups are more likely to form relationships [10]. According to the fact that the couples are intermarried within or outside the group, we can divide the mating pattern into two modes: Homogeneous marriage (i.e., endogamy) and heterogenous marriage (i.e., intermarriage) [11,12]. As one of the three major indicators of societal openness, mating pattern is not only related to the life course of individuals and the happiness of family life, but also to the macro-social consequences such as the distribution of social capitals, the opening of social strata constitute and reproduction of inequality [13,14,15,16,17,18,19,20], which will affect the long-term development of the population.

As early as the 1950s, western academia has studied the issue of mating pattern [21,22]. Kalmijn believes that the mate choice is a multidimensional phenomenon and makes a distinction between ascribed and achieved characteristics. Ascriptive status homogamy is measured by the similarity of couples from the perspective of intergenerational class reproduction with respect to their family backgrounds, such as family economic status, parental occupational class, race, religion, and so on. While the achieved dimension of status homogamy is measured by the similarity of spouses’ cultural resources from the perspective of the new generation. For example, individuals’ educational attainment, work experience, and so forth [23]. In the modernization theory of mating, there is some consensus among western scholars that the influence of antecedent factors on mating will be constantly weakened while the importance of autogenous factors will be enhanced, which is a subsequent influence of the popularization of education, the continuous development of society and the frequent regional mobility [12,23,24]. Chinese scholars also find that the proportion of autogenous marriage has increased significantly since the founding of the People’s Republic of China [25,26].

Culture of Chinese conventional agricultural society laid the foundation of preference for marriage: Ascriptive-characteristics matched [11]. However, with the development of social economy and educational expansion, Chinese nationals’ educational attainment has shown substantial increase [27]. The social scope and frequency of the new generation in the new era have greatly increased, and the concept of free love has been deeply rooted in people’s mind. In order to have the same interest, emotional resonance and commom goal, contemporary young people pay more and more attention to the homogeneity matching of their achieved dimension of status, especially the homogeneity of educational attainment. Many studies verify the rationality of educational assortative mating: it may improve marital satisfaction, maximize family output and enhance marriage stability [28,29,30]. Prior studies have demonstrated that education is a more important boundary in marriage selection than social-class origins and that educational homogamy has increased over time [23]. Some scholars attribute the increased degree of educational homogamy in China to the decreased gender gap in educational attainment [31,32].

It has been shown that marriage is good for health promotion [33,34], married people are more likely to have healthier behaviors than unmarried ones [34], have higher level of life satisfaction and stronger sense of well-being, with a lower risk of depression [35,36]. The selection of a mate has profound consequences for individuals. Socialization is a broad term used in this context to describe the impacts couples have on each other once they get married. The literature includes corresponding terms to explain the mechanism, including contagion, cohabitation effects, reciprocal exchange, social amplification, partner influence [37], and bargain power [38]. In the study of mating affecting health, some scholars in China found that hypergamy can inhibit female’s depression level and improve their life satisfaction while it has no effect on male’s life satisfaction and mental health [39]. Therefore, there is no doubt about the importance of research on mating mode.

The broad and profound connection between education and health has long been revealed [18,40,41]. In current research on the consequences of mating, many studies have found that marital education matching has an impact on people’s health. Multiple studies in the West [42,43,44,45] indicate that the husband’s risk of death will increase if his wife is more educated than himself. Yet some studies show that educational heterogeneity between couples will not affect their health [46]. Chinese scholars’ researches on the influence of mating on people’s health mostly focus on the field of mental health [19,20,39]. Some scholars have found that educational hypergamy can promote life satisfaction level of either gender [19]. It is worth noting that few studies in China have focused on the impact of marital matching patterns on people’s physical health.

As an important self-induced element in a decision or action to form an intimate relationship with another person, educational background can simultaneously reflect the individual’s socio-economic status and cultural capital [19]. In addition, it is more stable, reliable and easy to measure than occupation, income, reputation and other indicators [2]. On this basis, this study attempted to document the degree of educational assortative mating in Chinese internal migrants and how it evolves over time. We further analyzed the relationship between educational assortative mating and people’s self-rated health (SRH), as well as its gender heterogeneity.

## 2. Materials and Methods

The data used in this study were from the Volume A of the China Migrants Dynamic Survey (CMDS_A) designed and conducted by National Health Commission of the People’s Republic of China in 2017 (N = 169,989). Chinese who migrate from their hometown to other regions for at least one month without their *hukou* moving in are defined as the internal migrants (excluding students, soldiers and people whose couples are not migrants). A stratified, multi-stage and scale proportional Probability Proportionate to Size Sampling method is used, covering internal migrants aged over 15 years in 31 provinces (districts and cities) and Xinjiang production and Construction Corps in China.

Our study focused on the young and middle aged Chinese internal migrants who were first-married and conformed to the legal marriage age (Considering the current legal age for marriage in China was revised in 1980, this study used the legal age for marriage stipulated in Marriage Law of the People’s Republic of China (1950): Twenty for men and eighteen for women). The inclusion criteria for this study were as follows: (1) both the individuals and his/her spouse were first-married; (2) males were 20–59 years old and females were 18-59 years old. We focused on first-married couples for there might be differences between love and actual marriage, legal marriage and factual marriage, first marriage and remarriage in mate selection. The mixed use of the data of different marriage types may lead to an increase in confounding factors. Missing values on key variables were deleted using a list-wise method. A total of 127,829 samples were used in this study, including 65,416 males (51.2%) and 62,413 females (48.8%).

Using information on the educational attainment, the individuals and their spouses were assigned to one of seven mutually exclusive groups according to the highest level of education completed: Illiterate, Primary School, Junior Middle School, Senior Middle School, College, University and Postgraduate. If the respondent and his/her spouse shared the same educational attainment, he/she was in educational homogamy. If the respondent was less educated than his/her spouse, he/she was in educational hypergamy. If the respondent was more educated than his/her spouse, he/she was in educational hypogamy. D = E_respondent_ − E_respondent’s spouse_ was used to calculate the educational gap between the respondent and his/her spouse. E_respondent_ was the educational attainment of the respondent and E_respondent’s spouse_ was the respondent’s spouse’. According to the value of D, marital education matching pattern (M) was divided into three categories: educational homogamy (D = 0, coded as “0”), educational hypergamy (D < 0, coded as “−1”) and educational hypogamy (D > 0, coded as “1”).

SRH is a binary variable. Each individual was asked to rate his/her health status as “healthy”, “subhealthy”, “unhealthy, yet have the ability of self-care” and “lose the ability of self-care”. We considered those claimed to be healthy as healthy and others as unhealthy.

SPSS 20 and Stata 14 were used to process and analyze the data. SPSS 20 was mainly used for descriptive analysis, contingency table analysis, chi square test of key variables and logistic regression analysis (α = 0.05). Stata 14 was used for propensity score matching (PSM) analysis. PSM had been proved to be a useful, novel and creative statistical method when using non experimental data or observation data to evaluate the treatment effects [47]. In this method, the control variables were fitted to tendency values to match the individuals of the treatment group and the control group, so as to obtain more accurate average treatment effect. The model was constructed as follows:

(1) $$ATT=E\left(y_{k}-y_{0}|m=k\right)=E\left(y_{k}|m=k\right)-E\left(y_{0}|m=k\right)\;$$

In Formula (1), $y_{k}$ represented the SRH of the respondent whose pattern of marital education matching was k (= −1, 1), $y_{0}$ represented that of the respondent who was in educational homogamy (reference item). The model estimated the SRH difference of the internal migrants whose pattern of marital education matching was k between the state ($E\left(y_{k}│m\;=\;k\right)$) and its reference counterfactual state ($E\left(y_{0}│m\;=\;k\right)$) to obtain the net effect of different heterogenous educational mating patterns on the SRH of Chinese internal migrants.

## 3. Results

### 3.1. Demographic Characteristics

In Table 1, we documented key characteristics of the samples in this study. The results demonstrated that most individuals (72.9%) lived in urban areas while 79.2% were still registered in agriculture. 83.5% of the individuals had a job while the other did not. On average for all ages considered, most individuals were healthy (82.8%).

### 3.2. Educational Mating Structure and Its Changes in Chinese Internal Migrants

#### 3.2.1. Educational Homogamy Was Dominant in Chinese Internal Migrants

Table 2 shows the marginal distribution of the educational attainment of the first-married Chinese internal migrants and his/her spouse’s. From the table, we could find that the educational attainment of Chinese internal migrants was mainly concentrated in junior middle school degree. The proportion of educational homogamy (sum of diagonal cells in Table 2) in the respondents was 58.8%, the proportion of “male more educated” (sum of all cells below the diagonal in Table 2) was 27.2%, and “female more educated” (sum of all cells above the diagonal in Table 2) was 14.0%. The results in Table 2 indicated that “educational homogamy” and “male more educated” were the main marital education matching patterns in first-married couples of Chinese internal migrants. In addition, in heterogamy, the educational gap between the respondent and his/her spouse seldom exceeded two gradients (0.9%).

**Figure 1:**
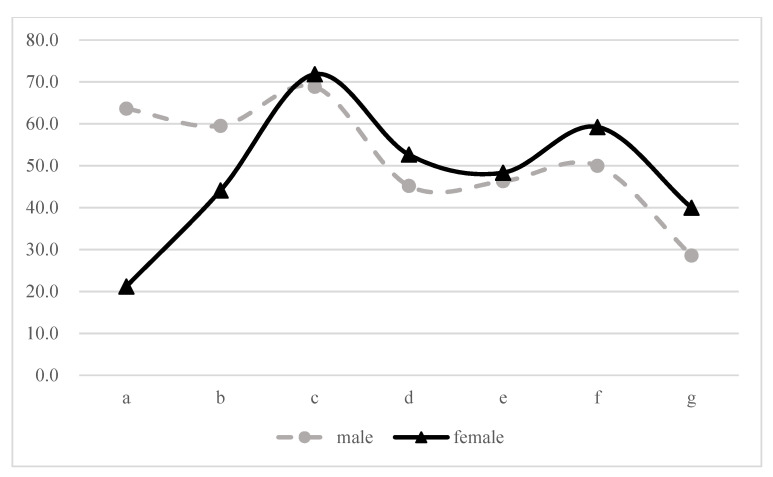
Proportion of homogamy in male and female at different levels of education (%)(N = 127,829). a. Illiterate, b. Primary School, c. Junior Middle School, d. Senior Middle School, e. College, f. University, g. Postgraduate.

Figure 1 shows the proportion of educational homogamy in male and female at different levels of education. The results showed that the proportion of educational homogamy in people with junior high school education were the highest both in male (68.8%) and in female (71.8%). There was an M curve correlation between the proportion of educational homogamy and educational level in both genders. Besides, in individuals with a junior high school degree or above, the proportion of educational homogamy in female is higher than that in male.

#### 3.2.2. Difference in Educational Matching Structure in Different Internal Migrants

Table 3 shows the educational matching structure in different internal migrants. As can be seen from the table, the education matching structure varied with individuals’ sex, community, household registration type, educational attainment, birth cohort, work status as well as whether they had a real estate (*p* < 0.001). Most people tended to choose “educational homogamy”. In the education heterogeneous marriage, the proportion of “educational hypergamy” was lower in male (14.0%) than that in female (27.9%), while the proportion of “educational hypogamy” was higher in male (26.6%) than that in female (13.9%). Among the internal migrants at different educational levels, the respondents at junior middle school level were more inclined to choose “educational homogamy” (71.4%), while that proportion in people with other educational attainment was less than 50%. The proportion of “educational homogamy” tended to increase over time.

#### 3.2.3. Educational Homogamy Increased over Time

**Figure 2:**
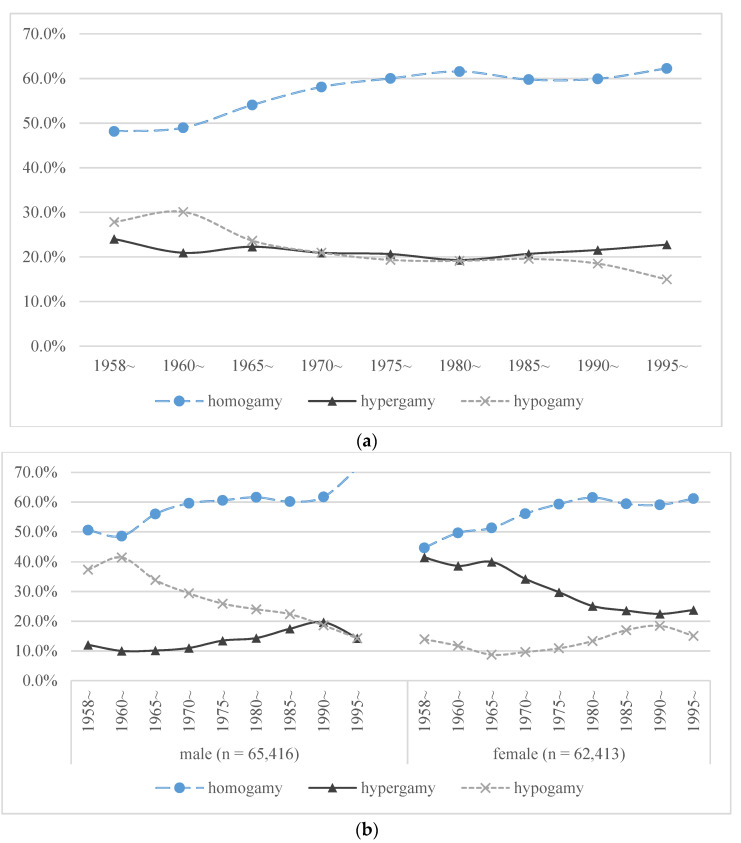
(**a**). Evolution of educational assortative mating with birth cohort (N = 127,829); (**b**). Evolution of educational assortative mating with birth cohort (Gender-specific outcomes).

In terms of different birth cohorts, there were significant differences in the marginal distribution of educational attainment of first-married Chinese internal migrants. Figure 2a shows the evolution of educational assortative mating in Chinese internal migrants of successive birth cohorts in 1958–1995. The proportion of educational homogamy increased from 48.1% to 62.3% within different birth cohorts, maintaining the dominant position in three patterns throughout. Educational hypergamy and educational hypogamy showed an upward, and downward trend, respectively after the birth cohort of reform and opening up (1980~ birth cohort). We further studied the evolution of educational assortative mating in Chinese internal migrants of different sexes. The results were shown in Figure 2b.

Figure 2b indicated that the proportion of educational homogamy had always occupied a dominant position in three patterns of educational matching modes in Chinese internal migrants of different sexes, and both showed an upward trend. However, it should be noted that in heterogeneous marriage, men were more likely to choose a partner with lower educational attainment than themselves, while women were more likely to choose a partner with higher educational attainment.

In general, the proportion of the two heterogeneous marriage patterns of men and women tended to converge in fluctuation over time. In precise, the proportion of educational hypergamy increased while the proportion of educational hypogamy decreased in male, and the proportion of educational hypogamy increased while the proportion of educational hypergamy decreased in female. This illustrated that the proportion of educational homogamy was increasing gradually, while the proportion of traditional gradient marriage (Male with better social status) was decreasing.

### 3.3. SRH of Internal Migrants

Table 4 shows the results of different Chinese internal migrants’ SRH status. It is illustrated that individuals’ SRH status might be related with their educational assortative mating, sex, community, household registration type, educational attainment and birth cohort (*p* < 0.001). Understanding whether they had a job or not, and whether they had a real estate with independent property rights, would also have an impact on their SRH (*p* < 0.001). Among Chinese internal migrants in different education matching patterns, the healthy proportion of “educational homogamy” was the highest (84.00%). The proportion of healthy people in male (83.60%) was slightly higher than that in female (82.00%). Results indicated that the healthy proportion in individuals would increase with their education level. Furthermore, the younger the birth cohort was, the higher the healthy proportion was. The healthy proportion in individuals who had a job was higher than that in the opposite ones (*p* < 0.001).

**Figure 3:**
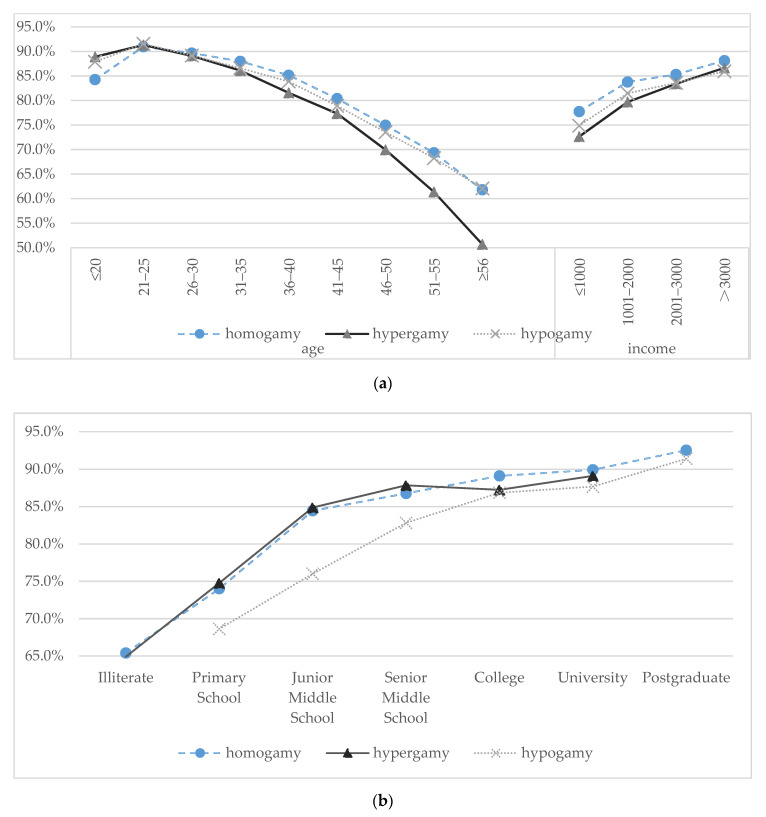
(**a**). SRH of different internal migrants (Age-specific and income-specific outcomes) (N = 127,829); (**b**). SRH of different internal migrants (Education-specific outcomes) (N = 127,829).

Figure 3a,b show the SRH status of different internal migrants. It can be observed from the figures that Chinese internal migrants’ SRH varied with their marriage patterns. Furthermore, there were health gaps between individuals in different marriage patterns, and the gaps would shift with their characteristics, such as age, income and educational attainment. Nevertheless, the health gap would gradually expand with age and narrow with education levels.

### 3.4. Educational Assortative Mating and Health

#### 3.4.1. Educational Hypogamy Would Impair the SRH of Chinese Internal Migrants

Table 5 shows the logistic regression results of some influencing factors on internal migrants’ SRH. Model 1 was the basic regression model to explore the influence of different matching modes on internal migrants’ SRH. Some factors (i.e., sex) were controlled for in Model 2–5 based on Model 1. Model 1 demonstrated that both educational hypergamy and educational hypogamy would reduce the probability of fair SRH. Specifically, internal migrants in educational hypergamy were 0.796 times (*p* < 0.001) more likely to think they were healthy than those in educational homogamy, and internal migrants in educational hypogamy were 0.858 times (*p* < 0.001) more likely to think they were healthy than those in educational homogamy. Yet the negative effects of educational hypergamy was not statistically significant in Model 3 after the respondent’s education was controlled for (*p* > 0.05). The negative impacts of educational hypogamy on internal migrants’ SRH remained statistically significant (all *ps* < 0.05) in Model 2–5.

We used PSM to explore the influence of different patterns of educational mating on the SRH of Chinese internal migrants. Results in Table 6 revealed no statistical differences in the probability of fair SRH between people in educational hypergamy and people in educational homogamy (*p* > 0.1). Compared with people in educational homogamy, people in educational hypogamy reduced the ratio of fair SRH by 4.1%, indicating that educational hypogamy would impair Chinese internal migrants’ SRH (*p* < 0.001).

#### 3.4.2. Gender Heterogeneity in Educational Assortative Mating

We further investigated the gender heterogeneity within the impact of educational assortative mating on Chinese internal migrants’ SRH by PSM. Results in Table 7 showed no statistical differences in the probability of fair SRH between people in educational hypergamy and people in educational homogamy (*p* > 0.1). Compared with people in educational homogamy, people in educational hypogamy reduced the ratio of fair SRH by 4.1% and 6.2%, indicating that educational hypogamy would impair the SRH of Chinese internal migrants (*p* < 0.001) and the impairment in the SRH of female was stronger than that of male.

## 4. Discussion

The results of this study indicated that degree of educational assortative mating in Chinese internal migrants is increasing over time, similar to the results of previous studies in other populations [48,49]. Additionally, “educational homogamy” and “traditional gradient marriage” were still the main marital education matching patterns in first-married couples of Chinese internal migrants. Even in heterogamy, the educational gap between the respondent and his/her spouse seldom exceeded two gradients. Except for the inclination of assortative mating [2], the equality of educational opportunities between men and women could also conduce to the increase of educational homogamy [31]. Increase in educational attainment has been a long-term trend since the establishment of the People’s Republic of China. Chinese government has actively facilitated the improvement of Chinese educational system and gender equality. The popularization of elementary education [50,51] and College Expansion [52,53] have effectively expanded educational opportunities for female, contributing to the rise in females education [50] and the narrowing of gender differences in education [31]. These macro-institutional changes may create a structural convenience for educational assortative mating.

In Chinese conventional agricultural society, families of different social strata typically identify that it would be better to choose a marriage between families of equal social rank and encourage their children to intermarry within their own classes. The establishment of this homogeneous marriage relationship can become a tool to maintain the existing social structure and enhance the unity within the class [22] and one of the main mechanisms of intergenerational transmission and reproduction [54]. With the development of industrial revolution and urbanization, regional mobility and social status mobility become more and more frequent. In modern society, the concept of free love and “love is the basis of marriage” have been deeply rooted among the people, and the traditional mate selection control means have been difficult to work. Therefore, the theory of modernization predicts that the characteristics of intra-class marriage in traditional marriage will decline under the impact of modernization. Our results indicated that the importance given to the homogeneity matching of individuals’ achieved characteristics in Chinese internal migrants’ mate selection has increased over time, which could represent improvements in social openness in China from the side.

The results of this study illustrated that educational hypogamy impair the SRH of Chinese internal migrants. The analysis of gender heterogeneity showed that the damage of educational hypogamy to the health of female was stronger than that of male. In other words, educational homogamy would be beneficial to promote individuals’ health. The fact that educational assortative mated individuals are healthier than others could be due to a lot of factors. In general, individuals who have a spouse in a higher class may enjoy increased health advantages than others as he/she can obtain higher material benefits and more social support in such marriage. Compared with individuals who are in educational hypogamy, individuals who marry an individual who has equal or better educational background than themselves may enjoy more health benefits for the connection between education and health [18,40,41]. However, based on the theory of maximizing the interest, Becker believes that “an efficient marriage market always has a perfect match”, and a well-matched marriage helps to make full use of social network resources of both sides [55]. Moreover, social maturity, companionship and spousal support from marriage are closely associated with the educational marriage matching pattern. According to social cognitive theory, similar educational attainment is conducive to optimal coordination of cognition and emotion, leading to the phenomenon of “likes attract likes” [56]. Positive emotion would improve people’s subjective well-being and further promote their SRH [57]. Consequently, educational assortative mating would facilitate Chinese internal migrants’ health related social capital and social support, and increase individuals’ life satisfaction, as well as their health status in sequence.

## 5. Conclusions

The popularization of elementary education and College Expansion in China have increased nationals’ educational attainment and promoted educational equality. The narrowed gender differences in education increases the degree of educational assortative mating in Chinese internal migrants in turn. Individuals’ achieved characteristics, especially their educational attainment, play an increasingly important role in mating, posing both opportunities and constraints in marriage market. “Likes attract likes” is not just about love, but also an important part of health. Education matching in marriage is an important factor, not only for the quality of marriage itself, but also for individuals’ health.

This study contributes specifically to the research on patterns of educational matching in marriage of Chinese internal migrants and its impact on their SRH. However, it has the following four limitations: First, due to permissions granted under the databank, this study lacks some variables such as marriage duration and people’s age married, which are important factors in the mechanism of marriage affecting health. Second, based on the distribution pattern of individuals’ SRH, we adopted a two-tier, binary health scale rather than differentiate the assessment of health, thus lost the possibility of indicating any intermediate levels. Third, in the study of the trend of marriage matching, only birth cohort was considered, while the effect of period should also be considered. Last but not least, though PSM are used for counterfactual inference, yet data we used are cross-sectional, tracking data may be better for causal inference.

## Figures and Tables

**Table 1:** Definition and Statistical Description of Major Variables (N = 127,829).

Variables	Variable Assignment/Range	Mean	SD
M	homogamy = 0; hypergamy = −1; hypogamy = 1	0.004	0.642
Community	urban = 1; rural = 2	1.271	0.444
Hukou	agriculture = 1; non-agriculture = 2	1.208	0.406
Age	18–59	37.239	8.712
Education	≤Primary School = 1; Junior Middle School = 2; ≥Senior Middle School = 3	2.192	0.709
Sex	male = 1; female = 2	1.488	0.500
Work	have a job = 1; have no job = 2	1.165	0.371
House	Self-owned = 1;Non-proprietary =2	1.691	0.462
Income	≤1000 = 1; 1001–2000 = 2; 2001–3000 = 3; >3000 = 4	2.996	0.271
SRH	Unhealthy = 0; Healthy = 1	0.828	0.377

**Table 2:** Comparison of educational level between husband and wife (%).

		Wife (N_2_ = 127,829)							Total _2_	
		a _2_	b _2_	c _2_	d _2_	e _2_	f _2_	g _2_		Homogamy _1_
**Husband (N_1_ = 127,829**)	a _1_	0.7	0.3	0.1	0.0	0.0	0.0	0.0	1.1	63.6
	b _1_	1.4	7.5	3.1	0.4	0.1	0.0	0.0	12.6	59.5
	c _1_	1.0	7.8	32.9	5.0	0.9	0.2	0.0	47.8	68.8
	d _1_	0.2	1.3	8.2	9.9	1.9	0.5	0.0	21.9	45.2
	e _1_	0.0	0.1	1.2	2.6	4.4	1.2	0.0	9.5	46.3
	f _1_	0.0	0.0	0.3	0.8	1.9	3.2	0.2	6.4	50.0
	g _1_	0.0	0.0	0.0	0.0	0.1	0.3	0.2	0.7	28.6
Total _1_		3.3	17.0	45.8	18.8	9.1	5.4	0.5	100.0	
Homogamy _2_		21.2	44.1	71.8	52.7	48.4	59.3	40.0		

Note: 1. The number in each cell was the sample proportion. Cells on the main diagonal showed the proportion of couples sharing the same educational attainment (homogamy) while the others showed the proportion of heterogamy. In heterogeneous marriage, the cells below the main diagonal showed the proportion of couples that wives were less educated than their husbands (male more educated) while the cells above the main diagonal showed the proportion of couples that wives were more educated than their husbands (female more educated). 2. a. Illiterate, b. Primary School, c. Junior Middle School, d. Senior Middle School, e. College, f. University, g. Postgraduate. Homogamy _1_, Homogamy _2_ showed the proportion of heterogamy in male and female at different educational levels respectively.

**Table 3:** Educational matching structure in different internal migrants (N = 127,829).

		Mating Patterns			χ^2^	*p*
		Homogamy	Hypergamy	Hypogamy		
Sex	male	59.4%	14.0%	26.6%	5483.304	<0.001
	female	58.3%	27.9%	13.9%		
Community	urban	58.3%	21.2%	20.6%	51.751	<0.001
	rural	60.4%	19.7%	19.9%		
*Hukou*	agriculture	59.8%	21.0%	19.2%	406.357	<0.001
	non-agriculture	55.2%	20.0%	24.8%		
Education	≤primary school	47.2%	48.5%	4.3%	25140.268	<0.001
	junior middle school	71.4%	16.3%	12.2%		
	≥senior middle school	48.5%	13.2%	38.3%		
Cohort	before 60 s	48.1%	24.0%	27.8%	565.260	<0.001
	after 60 s	52.4%	21.8%	25.7%		
	after 70 s	59.1%	20.8%	20.1%		
	after 80 s	60.6%	20.1%	19.4%		
	after 90 s	60.1%	21.7%	18.2%		
Work status	have a job	59.4%	19.4%	21.2%	868.310	<0.001
	have no job	56.0%	27.9%	16.2%		
House	self-owned	55.7%	23.2%	21.1%	274.776	<0.001
	non-proprietary	60.2%	19.7%	20.1%		

**Table 4:** SRH of different internal migrants (N = 127,829).

		SRH		χ^2^	*p*
		Unhealthy	Healthy		
M	homogamy	16.00%	84.00%	176.544	<0.001
	hypergamy	19.40%	80.60%		
	hypogamy	18.20%	81.80%		
Sex	male	16.40%	83.60%	55.333	<0.001
	female	18.00%	82.00%		
Community	urban	17.60%	82.40%	36.650	<0.001
	rural	16.10%	83.90%		
*Hukou*	agriculture	17.70%	82.30%	103.128	<0.001
	non-agriculture	15.10%	84.90%		
Education	≤Primary School	27.20%	72.80%	2074.445	<0.001
	Junior Middle School	16.50%	83.50%		
	≥Senior Middle School	13.30%	86.70%		
Cohort	before 60 s	43.50%	56.50%	4666.510	<0.001
	after 60 s	30.80%	69.20%		
	after 70 s	20.10%	79.90%		
	after 80 s	12.40%	87.60%		
	after 90 s	9.60%	90.40%		
Work status	have a job	15.90%	84.10%	740.179	<0.001
	have no job	23.60%	76.40%		
House	self-owned	18.50%	81.50%	70.445	<0.001
	non-proprietary	16.60%	83.40%		

**Table 5:** Influencing factors of internal migrants’ SRH (N = 127,829).

		Exp (B)				
Variable		Model 1	Model 2	Model 3	Model 4	Model 5
Mating	homogamy(Ref.)					
	hypergamy	0.796 ***	0.878 ***	0.974	0.809 ***	0.908 ***
	hypogamy	0.858 ***	0.884 ***	0.816 ***	0.956 *	0.877 ***
Sex	male(Ref.)					
	female		0.848 ***	0.862 ***	0.850 ***	0.859 ***
Age			0.945 ***	0.950 ***	0.950 ***	0.951 ***
Community	urban(Ref.)					
	rural		1.170 ***	1.206 ***	1.205 ***	1.209 ***
*Hukou*	agriculture(Ref.)					
	non-agriculture		1.196 ***	1.117 ***	1.110 ***	1.111 ***
House	self-owned(Ref.)					
	non-proprietary		1.046 **	1.070 ***	1.071 ***	1.071 ***
Work status	have a job(Ref.)					
	have no job		0.639 ***	0.640 ***	0.643 ***	0.641 ***
Income	≤1000(Ref.)					
	1001–2000		1.274 ***	1.223 ***	1.228 ***	1.218 ***
	2001–3000		1.369 ***	1.285 ***	1.291 ***	1.278 ***
	>3000		1.595 ***	1.474 ***	1.476 ***	1.466 ***
Range of migration	inter-provinces(Ref.)					
	cross-city within province		0.854 ***	0.848 ***	0.847 ***	0.847 ***
	cross-county within city		0.792 ***	0.780 ***	0.780 ***	0.778 ***
Duration of migration			0.989 ***	0.989 ***	0.989 ***	0.989 ***
E_respondent_	≤primary school(Ref.)					
	junior middle school			1.387 ***		1.274 ***
	≥senior middle school			1.520 ***		1.304 ***
E_respondent’s spouse_	≤primary school(Ref.)					
	junior middle school				1.300 ***	1.129 ***
	≥senior middle school				1.498 ***	1.209 ***

* *p* < 0.05, ** *p* < 0.01, *** *p* < 0.001.

**Table 6:** Effects of educational assortative mating on health (N = 127,829).

	SRH	Coef.	Robust SE	*p*	95% CI	
ATE						
	homogamy(ref.)	0.836	0.001	<0.001	0.833	0.838
	hypergamy	0.029	0.003	0.366	−0.003	0.009
	hypogamy	−0.041	0.006	<0.001	−0.052	−0.029

**Table 7:** Gender heterogeneity in impacts of educational assortative mating on SRH.

SRH		Male (N = 65,416)				Female (N = 62,413)			
		Coef.	*p*	95%CI		Coef.	*p*	95%CI	
ATE									
	homogamy(ref.)	0.844	<0.001	0.840	0.847	0.826	<0.001	0.822	0.830
	hypergamy	−0.003	0.578	−0.008	0.014	−0.002	0.539	−0.010	0.005
	hypogamy	0.041	<0.001	−0.053	−0.029	−0.062	<0.001	−0.088	−0.035

## Data Availability

Restrictions apply to the availability of these data. Data were obtained from Migrant Population Service Center, National Health Commission P.R. China and are available online at http://www.chinaldrk.org.cn with the permission of Migrant Population Service Center, National Health Commission P.R. China.

## Abbreviations

CMDS_A	the Volume A of China Migrants Dynamic Survey;
SRH	self-rated health;
PSM	propensity score matching;
CI	confidence interval
